# Solvent-induced assembly of mono- and divalent silica nanoparticles

**DOI:** 10.3762/bjnano.14.6

**Published:** 2023-01-06

**Authors:** Bin Liu, Etienne Duguet, Serge Ravaine

**Affiliations:** 1 Univ. Bordeaux, CNRS, CRPP, UMR 5031, 33600 Pessac, France; 2 School of Chemistry and Chemical Engineering, Liaocheng University, Liaocheng 252059, P. R. China; 3 Univ. Bordeaux, CNRS, Bordeaux INP, ICMCB, UMR 5026, 33600 Pessac, France

**Keywords:** assembly, chain stopper, patchy nanoparticles, patch-to-particle size ratio, self-assembly

## Abstract

Particles with attractive patches are appealing candidates to be used as building units to fabricate novel colloidal architectures by self-assembly. Here, we report the synthesis of one-patch silica nanoparticles, which consist of silica half-spheres whose concave face carries in its center a polymeric patch made of grafted polystyrene chains. The multistage synthesis allows for a fine control of the patch-to-particle size ratio from 0.23 to 0.57. The assembly of the patchy nanoparticles can be triggered by reducing the solvent quality for the polystyrene chains. Dimers or trimers can be obtained by tuning the patch-to-particle size ratio. When mixed with two-patch nanoparticles, one-patch nanoparticles control the length of the resulting chains by behaving as colloidal chain stoppers. The present strategy allows for future elaboration of novel colloidal structures by controlled assembly of nanoparticles.

## Introduction

Colloidal engineering has become an enormous research endeavor, with a major focus placed on creating increasingly scalable smart particles, such that desired structures can be assembled in a bottom-up fashion [1–5]. Among all the existing synthetic routes permitting to imbue functionality into a colloidal suspension, those dedicated to the formation of patchy particles have received particular attention [6–10]. Indeed, several generic models including the extended Kern and Kern-inspired patchy models [11–13], spot-like patchy models [14–15], and rigid-body patchy models [16–17] have shown a great potentiality of patchy particles to be used as building blocks for the assembly of a great variety of colloidal structures. Depletion interactions have been experimentally utilized to drive particles with a spherical cavity and complementary microspheres to form colloidal clusters [18]. Colloidal chains have been obtained by the assembly of Janus particles with one face selectively functionalized with DNA containing a self-complementary sticky end [19], particles with two patches functionalized with metal-coordination-based recognition units [20], and by co-assembly of block copolymer micelles and hard nanoparticles [21]. Particles with two patches located at opposite poles have been assembled into a Kagome lattice by hydrophobic interactions [22], into chains by liquid bridging [23], and into a series of structures under an AC electric field [24]. The linear self-assembly of patchy gold nanorods tethered with hydrophobic polymer chains at both ends can be triggered by solvophobic attractions induced by a change in solvent quality [25]. By using post-assembly ligand photo-cross-linking [26] or by adding monofunctional nanospheres into a suspension of bifunctional gold nanorods [27], it has been shown that the average degree of polymerization of the resulting chains can be controlled. The reduction of solvent quality may also be employed to induce the assembly of silica/polystyrene (PS) dumbbells [28] and silica nanoparticles with two PS patches (2-PSN) [29–31] into multipod-like clusters and colloidal chains, respectively. We have recently reported that the same strategy can be used to assemble one-patch silica nanoparticles (1-PSN) with a well-controlled patch-to-particle size ratio (PPSR) into dimers, trimers, tetramers, and spherical micelles at a low incubation time in mixtures of tetrahydrofuran (THF) and ethanol [32]. Here, we extend the study to 1-PSN with smaller PPSR values and to the use of another poor solvent for the PS patch (i.e., salty water). We show that only dimers or trimers can be obtained due to steric hindrance induced by the large silica cap of the patchy nanoparticles. The present study also extends the insights we recently gained about the capability of using 1-PSN with a PPSR of 0.60 as chain stoppers [31]. We show that the addition of 1-PSN with a lower PPSR value of 0.38 allows us to control the length of 2-PSN chains in a wider range of compositions.

## Results and Discussion

### Synthesis of one-patch silica particles with well-controlled patch-to-particle size ratio

**Figure 1:**
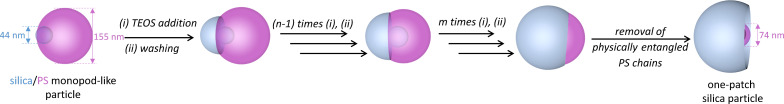
Synthetic route for the preparation of 1-PSN with a controlled patch-to-particle size ratio.

**Figure 2:**
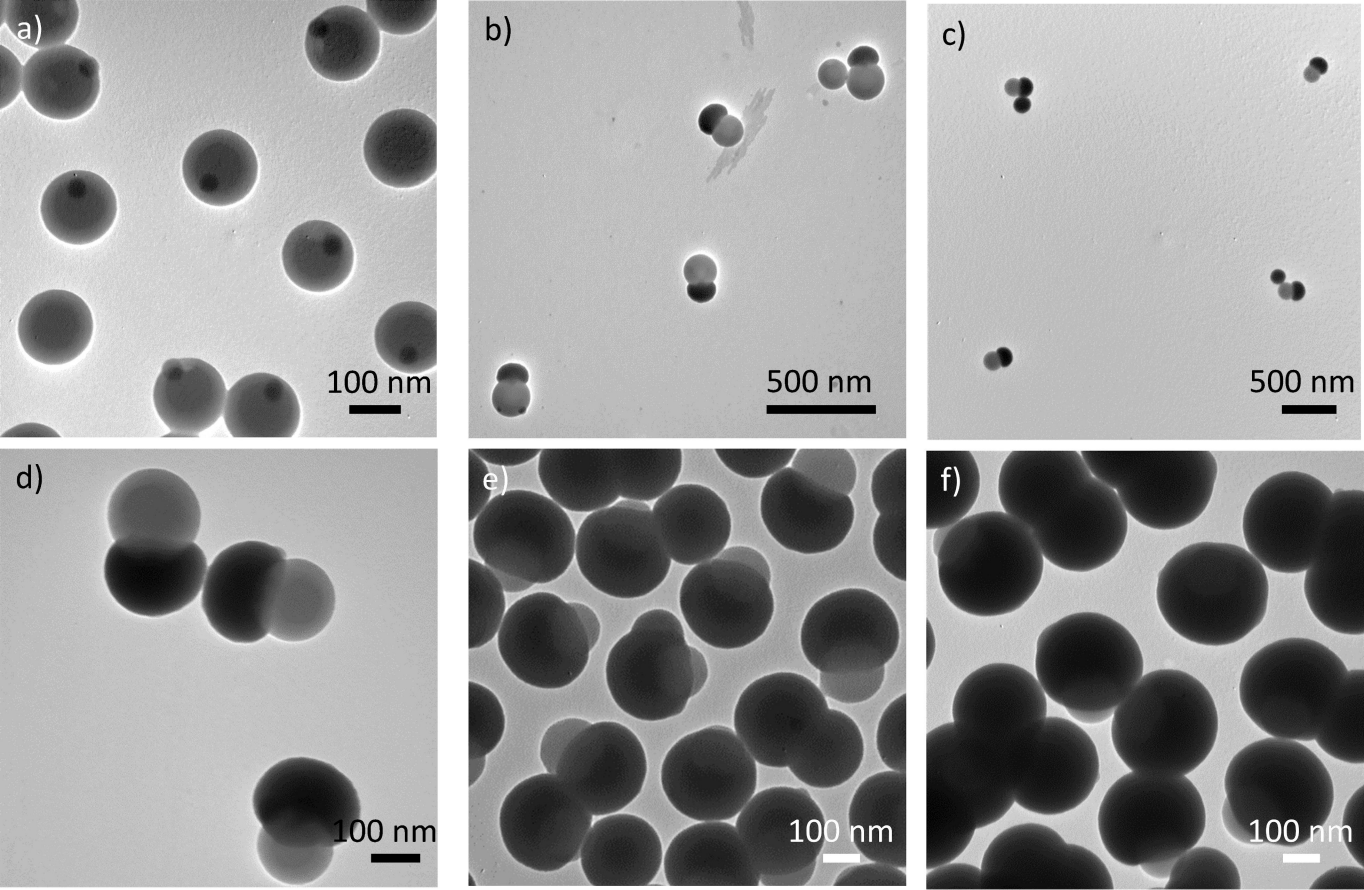
TEM images of the silica/PS monopods after (a) 0, (b) 1, (c) 2, (d) 4, (e) 9, and (f) 14 iterative silica growth steps.

Figure 1 shows the multistep approach developed to synthesize 1-PSN with controlled patch size. First, silica/PS monopods consisting of a central silica core attached to one PS nodule (Figure 2a) have been prepared by seeded-growth emulsion polymerization of styrene, as reported elsewhere [32] (see experimental details). The silica core of the silica/PS monopods was regrown through successive additions of a small amount of tetraethoxysilane (TEOS) interspersed with centrifugation/redispersion cycles in order to avoid the occurrence of secondary nucleation of silica [30]. Figure 2b–f show the morphology of silica/PS nanoparticles after 1, 2, 4, 9, and 14 iterative silica growth steps, with measured diameter of the silica cap of 130, 165, 195, 250, and 315 nm, respectively. The selective dissolution of the physically entangled PS chains by three centrifugation/redispersion cycles in 20 mL of THF was thus performed to get the 1-PSN with controlled PPSR, which is defined as the ratio of the diameter of the PS patch divided by the diameter of the silica cap.

**Figure 3:**
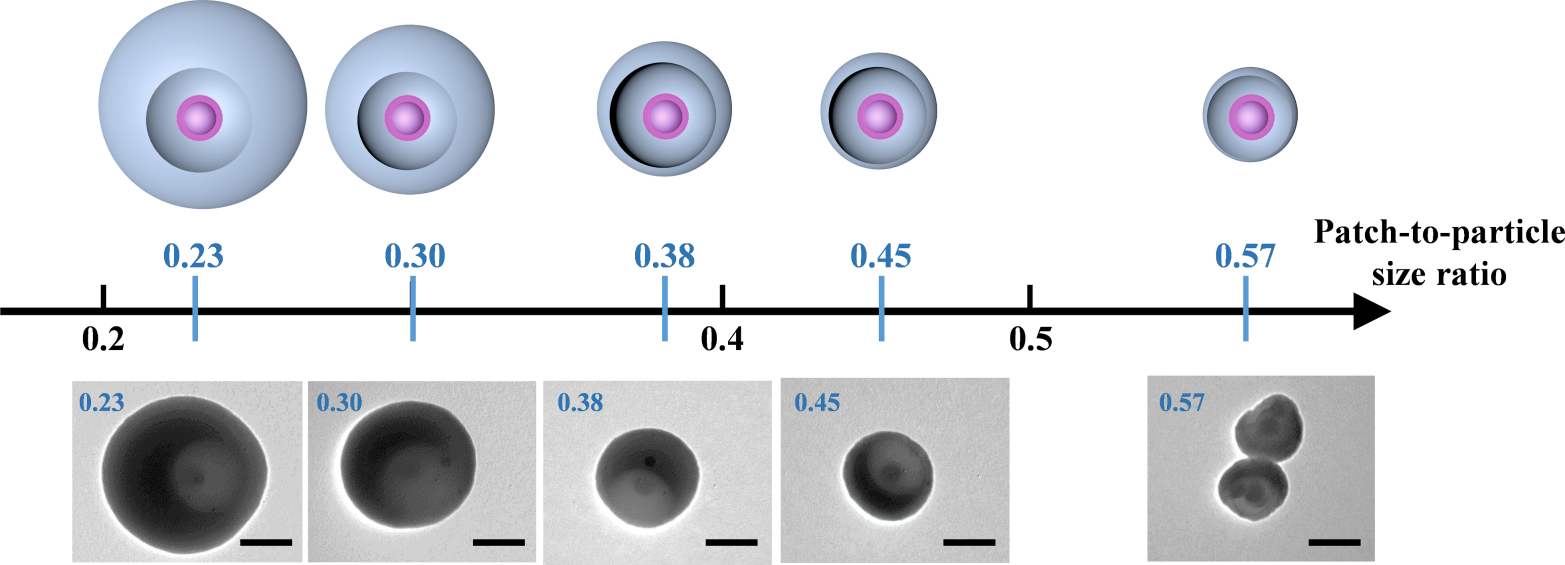
Schemes and representative TEM images of 1-PSN with PPSR varying from 0.23 to 0.57. Scale bars: 100 nm.

As shown in Figure 3, this led to the formation of silica particles with one circular cavity, at the bottom of which the accessible surface of the initial silica seed is decorated by a PS shell of approx. 15 nm (as estimated by transmission electron microscopy, TEM). The latter is made up of covalently grafted PS macromolecules resulting from the copolymerization of styrene with methacryloxymethyl groups. As previously demonstrated [25], these PS chains can serve as sticky patches when their solubility is reduced through the addition of a poor solvent. Therefore, the diameter of the patch is approx. 74 nm and the PPSR of the as-obtained 1-PSN vary from 0.23 to 0.57 (Figure 3).

### Influence of the patch-to-particle size ratio on the self-assembly behavior of one-patch silica particles

**Figure 4:**
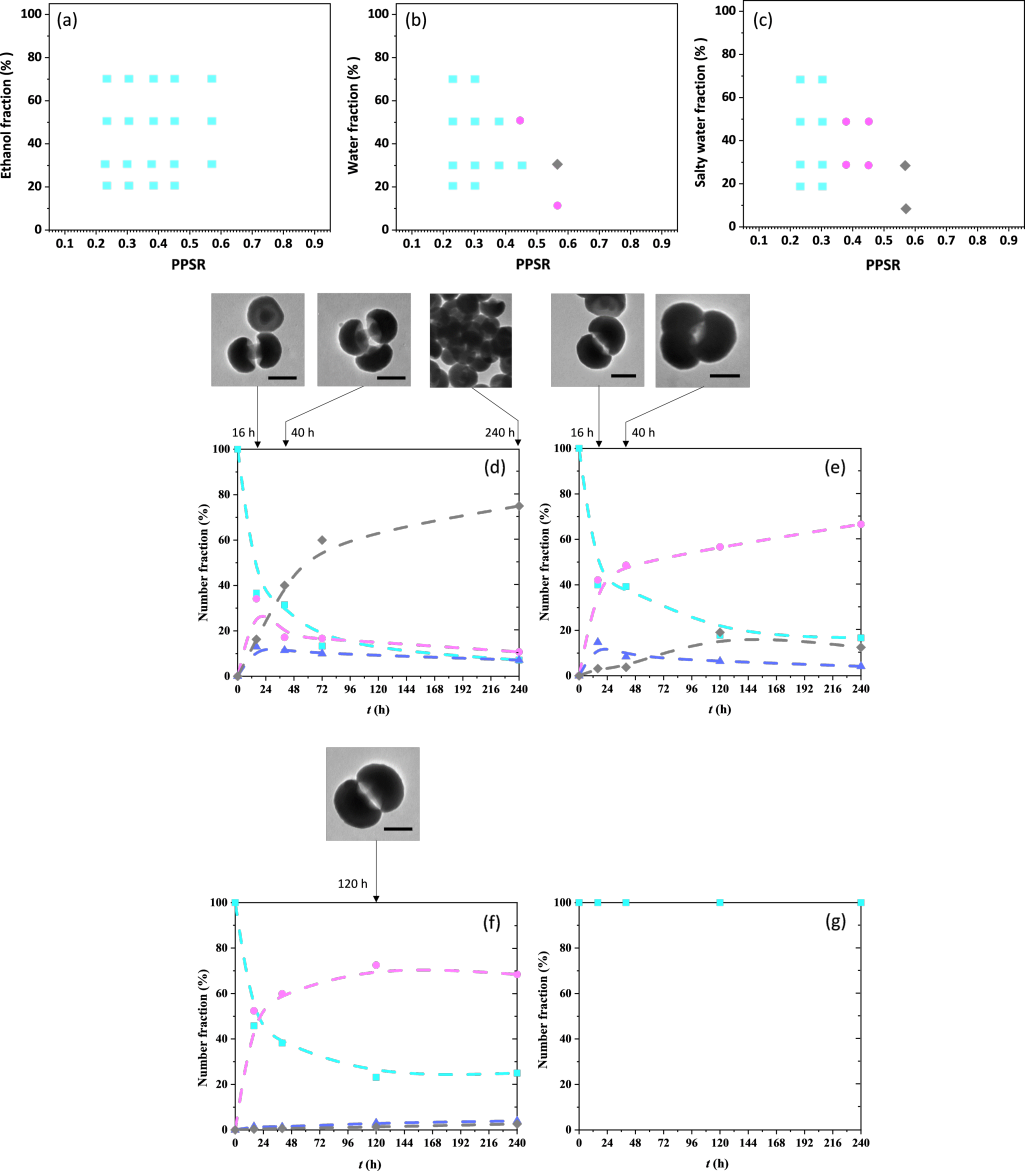
Phase diagrams identifying the main products of self-assembly (1-PSN (light blue squares), dimers (magenta circles), and large clusters (grey diamonds)) as a function of the patch-to-particle size ratio of 1-PSN in different solvent mixtures after 10 days of incubation at room temperature: a) THF/ethanol, b) THF/water, and c) THF/salty water ([NaCl] = 20 mmol/L). Evolution with incubation times in a 7:3 (vol/vol) THF/salty water mixture of the fractions of 1-PSN (light blue squares), dimers (magenta circles), trimers (blue triangles), and large clusters (grey diamonds), as determined by statistical analysis of TEM images for different PPSR values: d) 0.57; e) 0.45; f) 0.38; g) 0.30 or 0.23. Dotted lines are a guide to the eye. Representative TEM images of dimers, trimers, and large aggregates formed after 16, 40, 120, or 240 h are shown on the top. Scale bars: 100 nm.

Our previous work on the assembly of 1-PSN with larger PPSR ranging from 0.69 to 1.54 in a THF/ethanol mixture has shown that colloids with a low aggregation number (e.g., dimers, trimers, tetramers, and spherical micelles) could be obtained at a low incubation time. Also, the higher the PPSR value, the more these micelles evolve into larger objects over long incubation periods. They elongated in the form of chains for PPSR = 0.87 or extended into bilayers for PPSR = 1.18 or 1.54 [32]. Here, we first tried to assemble 1-PSN with PPSR ranging from 0.23 to 0.57 in THF/ethanol mixtures. Figure 4a shows that all of them were found to be unable to self-assemble in the presence of ethanol, meaning that ethanol is not a sufficiently poor solvent for PS to make the patches attractive enough and/or the macromolecular interactions strong enough to maintain the assembly. We performed other series of experiments by using pure water (Figure 4b) or salty water (Figure 4c) instead of ethanol in different fractions. We observed that, except for 1-PSN with PPSR ≤ 0.3, assembly was possible and led to dimers and possibly to clusters of low aggregation number for the highest PPSR values. These results first indicate that the size of the silica cap of the 1-PSN has a strong influence on their capability to self-assemble. Indeed, when the regrown silica cap reaches a certain size, the PS chains which are grafted onto the initial silica seed surface and whose average mass was estimated to be approx. 500 000 g·mol^−1^ [33], can no longer interact because they are too far from each other. A closer examination of Figure 4b,c shows that water, and even more so salty water, is more efficient than ethanol to promote 1-PSN assembly. This is probably because salty water reduces both the electrostatic repulsions between nanoparticles due to negatively charged silanolate groups at their surface and also the solvent quality for the PS macromolecules. Minimization of the free surface energy of the system thus corresponds to the formation of physical bonds between the nanoparticles [34–35], which behave as sticky patches. The kinetics of the assembly in a 7:3 (vol/vol) THF/salty water mixture was more deeply investigated. Figure 4d shows that the incubation of 1-PSN with a PPSR of 0.57 led to the formation of dimers, trimers, and larger aggregates, whose number fraction after 10 days of incubation was 10%, 8%, and 74%, respectively. Interestingly, the incubation of 1-PSN with progressively smaller PPSR led to the formation of assemblies made of fewer and fewer 1-PSN (Figure 4e–g). More explicitly, only dimers and trimers were obtained from 1-PSN with a PPSR of 0.45, while only dimers were obtained when PPSR was 0.38.

**Figure 5:**
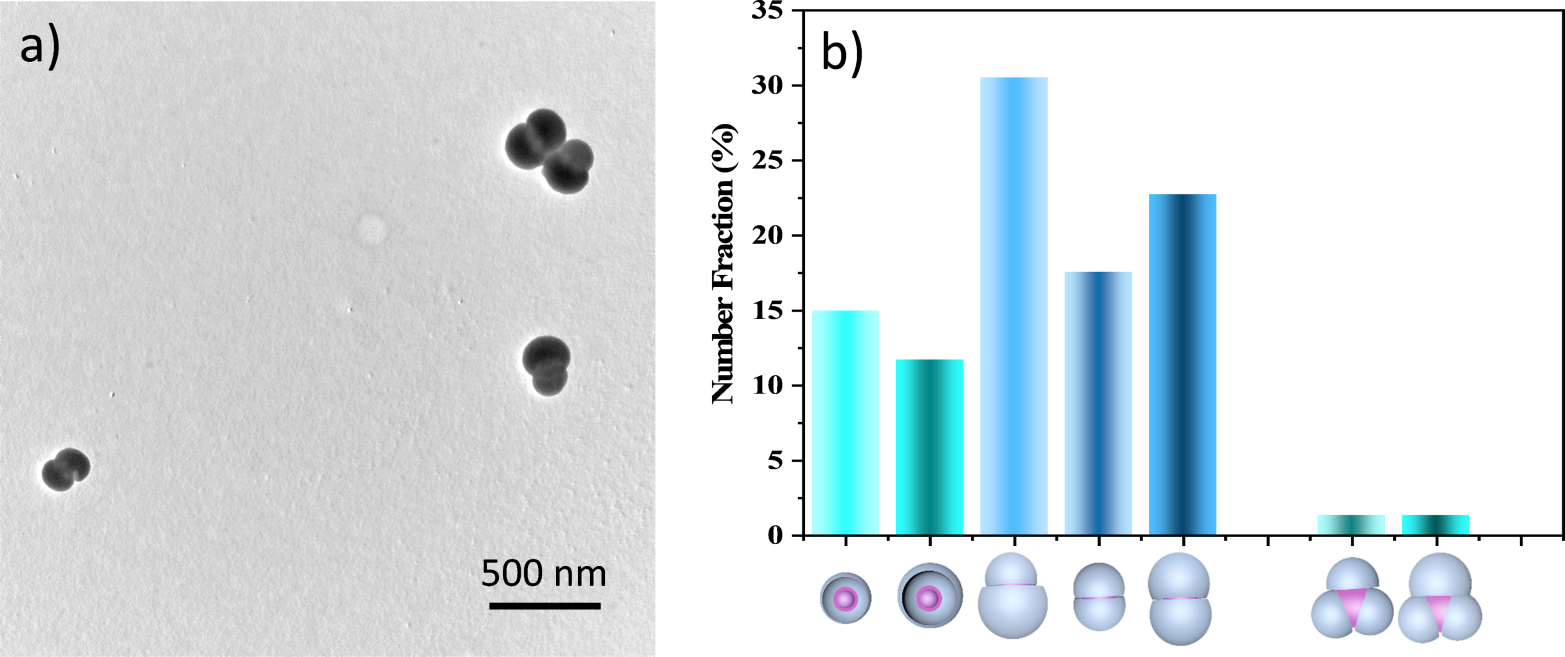
a) TEM image showing some heterodimers obtained after incubation in a 7:3 (vol/vol) THF/salty water mixture during 24 h of a 1:1 mixture of 1-PSN with a PPSR of 0.45 and 0.38. b) Number distribution of 1-PSN of both sizes, homo- and heterodimers, homo- and heterotrimers present in the incubation medium after 24 h, as determined by statistical analysis of TEM images.

The stickiness of the PS chains in the presence of salty water was further exploited by mixing 1-PSN of two different PPSR. The aim here was not to get heterodimers (i.e., resulting from the assembly of two different 1-PSN) with a high yield since homodimers could also be formed, but rather to perform a proof-of-principle experiment to demonstrate that such complex structures could be obtained. Figure 5a shows that heterodimers were effectively obtained by mixing 1-PSN with a PPSR of 0.45 and 0.38. After 24 h, the incubation medium consists of 1-PSN (≈26%), heterodimers (≈30.5%), homodimers made of two 1-PSN with a PPSR of 0.45 (≈17.5%), homodimers made of two 1-PSN with a PPSR of 0.38 (≈23%), and also heterotrimers (≈1.5%), and homotrimers (≈1.5%) (Figure 5b).

### Co-assembly of 2-PSN and 1-PSN acting as chain stoppers

**Figure 6:**
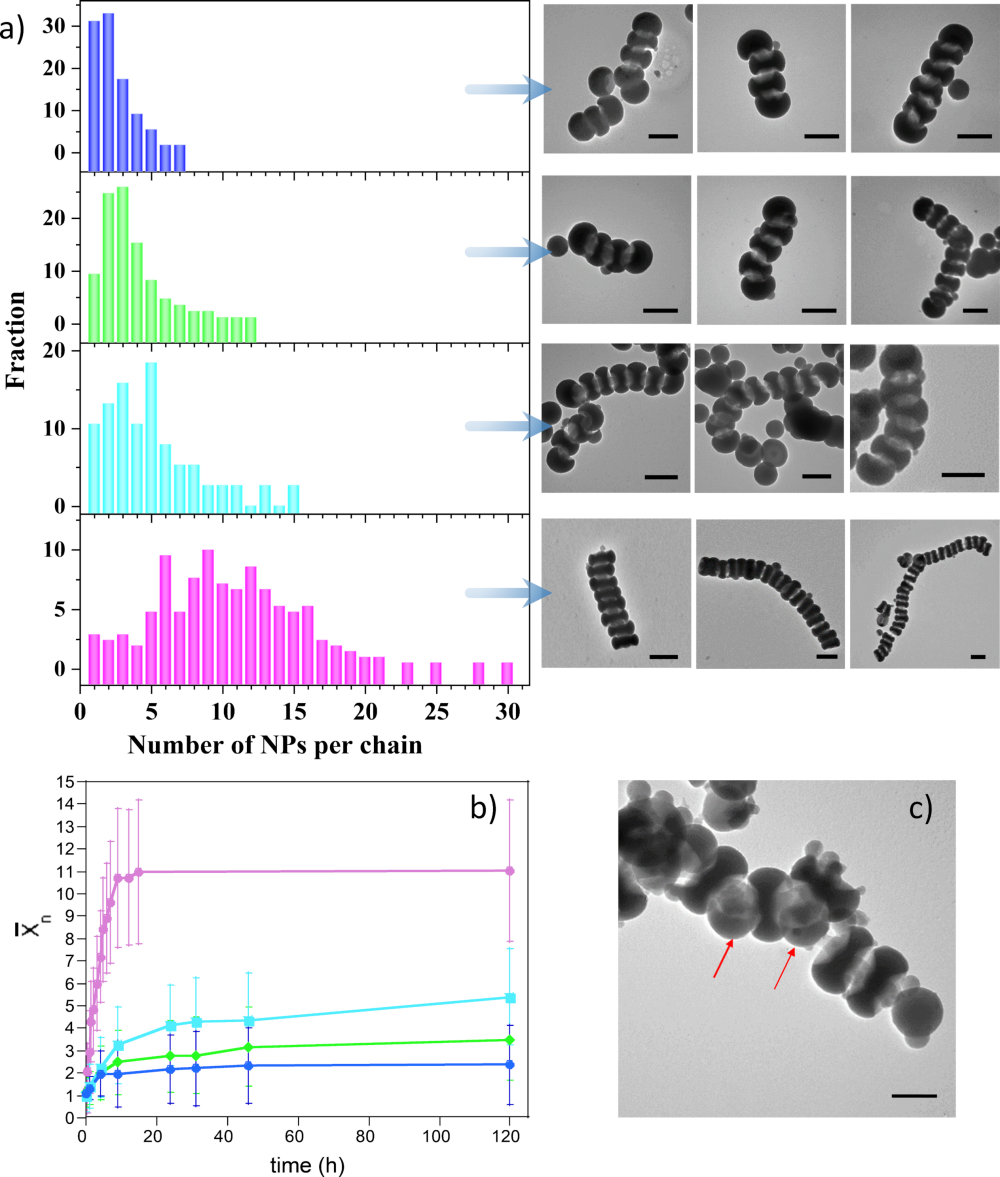
a) Chain length distribution and representative TEM images of chains for 1-PSN/2-PSN = 0 (magenta), 0.5 (light blue), 1 (green), and 1.5 (blue) at *t* = 120 h. Scale bars: 200 nm. Experimental conditions are displayed in Table 1. b) Time dependence of 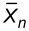 for 1-PSN/2-PSN = 0 (magenta), 0.5 (light blue), 1 (green), and 1.5 (blue). Curves are a guide to the eye. c) TEM image of a chain obtained by mixing 2-PSN with 1-PSN with a PPSR of 0.60 (1-PSN/2-PSN = 0.5; incubation time = 16 h). Scale bar: 100 nm.

It has been previously shown that monofunctional nanoparticles can act as chain stoppers, as their addition into suspensions of soft patchy nanoparticles [36], gold nanorods [27], or silver nanoplates [37] allowed for the control of chain length. We thus studied the capability of 1-PSN to act as chain stoppers when they are mixed with 2-PSN, whose solvent-induced assembly into colloidal polymers was recently reported [29–30]. Indeed, we previously showed that 2-PSN, which exhibit a disk-like morphology with a diameter of 190 nm, formed chains as long as 6 µm (Figure 6a, bottom row) when 30 vol % of salty water was added into the NP dispersion in THF. As a result, both electrostatic repulsions between NPs were reduced due to negatively charged silanolate groups at their surfaces and the solvent quality for the PS chains [29]. The statistical analysis of the TEM images obtained from samples collected at different incubation times allowed us to plot the time evolution of the average degree of polymerization, 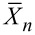, defined as:


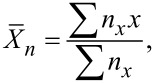


where *x* is the number of 2-PSN in the chain and *n_x_* is the number of chains containing *x* 2-PSN (Figure 6b, magenta curve). The linear relationship, which can be observed at a short incubation time (*t* < 2 h), is characteristic of a reaction-controlled step-growth polymerization, in which the reactivity of the patches is independent of the chain length [38]. At longer times (*t* > 2 h), one can observe that 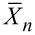 no longer varies linearly with time (Figure 6b). The polymerization of 2-PSN seems to follow a different pathway that is, most likely, a “diffusion-controlled” stage, which can be attributed to the fact that the sedimentation of the chains becomes the rate-limiting factor when they become relatively long. We recently reported that the length of the chains made of 105 nm or 130 nm 2-PSN can be controlled by the addition of 1-PSN with a PPSR of 0.60, which act as colloidal analogues of chain stoppers, at several [1-PSN]/[2-PSN] ratios between 0 and 0.5 [31]. Here we decided to further explore this behavior by expanding the range of [1-PSN]/[2-PSN] ratios from 0 to 1.5. We thus first tried to co-assemble 190 nm 2-PSN with 1-PSN with a PPSR of 0.60. Figure 6c shows that even if a small proportion of chains are capped with 1-PSN, most of these ones are interspersed between 2-PSN within the chains (red arrows), probably due to their small size. Assuming that the PPSR of 1-PSN should be at least equal to that of 2-PSN, so that they can play the role of chain stoppers, we thus decided to work with 1-PSN with a smaller PPSR of 0.38. Figure 6a shows that this time the 1-PSN are effectively located at both ends of the 2-PSN chains. In agreement with our previous results [31] and those reported by others [27,37], Figure 6a shows that the chain length strongly depends of the 1-PSN/2-PSN ratio: the higher the ratio, the shorter the chains after 120 h of incubation. The chain stopper strategy allowed us to finely tune the degree of polymerization at any incubation time, as shown in Figure 6b. For instance, at *t* = 9 h, the average number of 2-PSN in the chains is 10.7 in the absence of 1-PSN, whereas it is only equal to 3.2, 2.5, and 1.9 when the ratio 1-PSN/2-PSN is 0.5, 1, and 1.5, respectively.

## Conclusion

Following a multistep approach based on the conformational enlargement of the silica core of silica/PS monopods that we recently developed [30–32], one-patch silica nanoparticles with a controlled patch-to-particle size ratio ranging from 0.23 to 0.57 were synthesized. The decrease of the solvent quality for the PS patch induced by the addition of salty water made it sticky, which induced the assembly of the patchy silica nanoparticles into mostly dimers when the interactions between the PS chains were not annihilated by steric hindrance between the silica parts. We also showed that these one-patch silica nanoparticles can act as colloidal chain stoppers when mixed with divalent nanoparticles. We expect these results will inspire the fabrication by self-assembly of yet inaccessible colloidal structures. Bridging functionality at the colloidal chain end being now conceivable, their assembly into block-copolymer analogues can for instance be considered.

## Experimental

### Materials

Styrene (Sigma-Aldrich, 99%), methacryloxymethyltriethoxysilane (MMS, ABCR, 98%), sodium persulfate (Sigma-Aldrich, 99%), Symperonic^®^ NP30 (Aldrich), sodium dodecyl sulfate (SDS, Sigma-Aldrich, >90%), tetraethoxysilane (Sigma-Aldrich, 99%), ʟ-arginine (98.5%, Sigma-Aldrich), ammonium hydroxide (28–30% in water, SDS), sodium chloride, (≥99.0%, Sigma-Aldrich), and sodium hydroxide (≥98%, pellets, Sigma-Aldrich) were used as received. Ultrapure water with a resistivity of 18.2 MΩ·cm at 25 °C obtained from a Milli-Q system (Millipore) was also systematically used. Absolute ethanol and tetrahydrofuran (>99%) were purchased from VWR Chemicals.

### Synthesis of silica/PS monopods and bipods

In a manner similar to the already reported procedure [32], monopods consisting of a central silica core attached to one PS nodule have been prepared by seeded-growth emulsion polymerization of styrene. Briefly, silica nanoparticles with an average diameter of 44 ± 2 nm were obtained by TEOS hydrolysis/polycondensation according to a two-stage protocol. At the end of the synthesis, the silica surface was functionalized with methacryloxymethyl functions by reacting with MMS at room temperature for 3 h and then for one more hour at 90 °C under stirring. The added amount of MMS corresponded to a nominal grafting surface density of 0.7 funct./nm^2^. Then, MMS-functionalized silica NPs (1.8 × 10^16^ part/L) were used as seeds for the seed-growth emulsion polymerization of styrene (100 g/L) stabilized by a mixture (3 g/L) of Symperonic^®^ NP30 and SDS (5 wt %) and initiated by 1.3 mL of Na_2_S_2_O_8_ (0.1 g dissolved in 4 mL water) at 70 °C for 6 h to obtain silica/PS monopods with a PS pod diameter of about 155 nm. The morphological yield was 99%.

Bipods consisting of a central silica core surrounded by two PS nodules with a diameter of approx. 160 nm were obtained with a yield of 97% in a similar way from 55 ± 2 nm silica nanoparticles which were functionalized with MMS at 0.5 funct./nm^2^.

### Controlled growth of the silica core

According to the procedure reported in [30], 9.1 mL of absolute ethanol, 0.7 mL of ammonia, and 0.2 mL of the dispersion of monopods (1.8 × 10^16^ part/L) or bipods (1.8 × 10^16^ part/L) were introduced into a 25 mL flask and the mixture was homogenized using a magnetic bar. A volume of 200 µL of TEOS was added all at once after 5 min. The reaction was kept under stirring at 20 °C for 15 min. The reaction medium was poured into a 50 mL Falcon tube containing 15 mL of absolute ethanol. After 2 cycles of centrifugation (12,000*g;* 5 min) and redispersion in absolute ethanol, the nanoparticles were finally redispersed in 10 mL of a previously prepared hydroalcoholic solution (absolute ethanol/ammonia/water, volume ratio: 91%:7%:2%). This protocol was renewed to obtain the next generation. The final diameter of the silica core of the bipods was 190 nm.

### Dissolution of the PS nodules

For dissolving the PS nodules of the monopods and bipods, three centrifugation/redispersion cycles in 20 mL of THF (12,000*g*; 10 min) were performed. The concentration of 1-PSN and 2-PSN dispersions was adjusted to 1.08 × 10^15^ part/L and the solution was stored at 4 °C.

### Assembly of one-patch silica nanoparticles

The incubation of the nanoparticles was carried out in 15 mL tubes under rolling motion at 60 rpm at room temperature. A calculated volume of ethanol, water, or salty water (20 mM of a NaCl aqueous solution) was added dropwise under stirring into the THF dispersion of 1-PSN to reach the targeted volume fraction and a total volume of 1 mL. It took about 20 s to add 100 μL. Assembled structures were monitored by collecting 50 µL samples at various incubation times and direct deposition on TEM grids.

### Co-assembly of one- and two-patch silica nanoparticles

The incubation of the nanoparticles in a 7:3 (vol/vol) THF/salty water mixture was carried out in 15 mL tubes under rolling motion at 60 rpm and at room temperature. The composition of the mixtures is given in Table 1.

**Table 1:** Experimental conditions used for the co-assembly of 2-PSN and 1-PSN.

1-PSN/2-PSN particle ratio	volume (µL)			

	1-PSN dispersion	2-PSN dispersion	THF	salty water

0	0	700	1400	900
0.5	350	700	1050	900
1	700	700	700	900
1.5	1050	700	350	900

### Characterization methods

Transmission electron microscopy experiments were performed using a Hitachi H600 microscope operating at an acceleration voltage of 75 kV. The samples were prepared by depositing one drop of the colloidal dispersion on conventional carbon-coated copper grids. The liquid evaporated in the open air at room temperature and the grids were placed in a box protected from dust. Statistics from image analysis were performed over at least 300 multipods or 200 chains. The ζ potential value of 1-PSN aqueous dispersions (pH ≈5.7) was measured using the Malvern Zetasizer 3000 HS setup (Malvern Instruments). The dielectric constant of water was set to 80.4 and the Smoluchowsky constant *f*(ka) was 1.5.
